# Sex Differences in Adverse Reactions to an Inactivated SARS-CoV-2 Vaccine Among Medical Staff in China

**DOI:** 10.3389/fmed.2021.731593

**Published:** 2021-09-08

**Authors:** Jian-Sheng Zhu, Mei-Xian Zhang, Ching-Wen Chien, Wei-Ying Yang, Gui-Feng Shi, Shulin Qiu, Tao-Hsin Tung, Hai-Xiao Chen

**Affiliations:** ^1^Department of Infectious Diseases, Taizhou Hospital of Zhejiang Province Affiliated to Wenzhou Medical University, Linhai, China; ^2^Evidence-Based Medicine Center, Public Laboratory, Taizhou Hospital of Zhejiang Province Affiliated to Wenzhou Medical University, Linhai, China; ^3^Institute for Hospital Management, Tsing Hua University, Shenzhen, China; ^4^Department of Intensive Care Unit, Taizhou Hospital of Zhejiang Province Affiliated to Wenzhou Medical University, Linhai, China; ^5^Department of Preventive Health Care, Taizhou Hospital of Zhejiang Province Affiliated to Wenzhou Medical University, Linhai, China; ^6^Institute for Hospital Management, Tsinghua University, Shenzhen Campus, Shenzhen, China; ^7^Department of Orthopedics, Taizhou Hospital of Zhejiang Province Affiliated to Wenzhou Medical University, Linhai, China

**Keywords:** adverse reaction, sex difference, SARS-CoV-2, COVID-19, inactivated vaccine

## Abstract

**Objective:** We investigated whether there were sex differences in adverse reactions to an inactivated SARS-CoV-2 vaccine among medical staff in China.

**Methods:** From 24 February to 7 March 2021 an online cross-sectional survey was conducted with a self-administered COVID-19 vaccine questionnaire among medical staff in Taizhou, China. In total, 1397 interviewees (1,107 women and 290 men) participated in the survey.

**Results:** In our study, 178 (16.1%) women and 23 (7.9%) men reported adverse reactions following their first vaccination, and 169 (15.3%) women and 35 (12.1%) men reported adverse reactions following their second vaccination. After adjusting for confounding factors, adverse reactions to other vaccines, worry about adverse reactions, knowledge of the inactivated vaccine being used in the hospital, taking the vaccine for one's family proactively and receiving an influenza vaccination were significantly related to adverse reactions to both injections in women. In contrast, in men, concerns about adverse reactions independently increased the risk of adverse reactions following either vaccination, and a history of adverse reactions to other vaccines also increased the risk of adverse reactions to both injections.

**Conclusions:** Sex differences in the frequency of reported adverse reactions to an inactivated SARS-CoV-2 vaccine and potential factors were demonstrated in a sample of medical staff.

## Introduction

Coronavirus disease 2019 (COVID-19) has swept across the world since the discovery of the novel coronavirus in Wuhan in December 2019. Vaccination to prevent severe acute respiratory syndrome coronavirus (SARS-CoV-2) infection is considered the most promising approach for controlling the pandemic. As of 1 June 2021, at least 13 different vaccines (across four platforms) have been administered, and six different vaccines have been listed for WHO Emergency Use Listing (EUL) (1), including the Pfizer/BioNtech Comirnaty vaccine, the AstraZeneca vaccine (AZD1222), the Janssen vaccine (Ad26.COV 2.S), the Moderna COVID-19 vaccine (mRNA-1273), the Sinopharm vaccine, and the Sinovac-CoronaVac. These vaccines have been demonstrated to be safe and efficacious. In China, two inactivated virus vaccines (the Sinopharm vaccine and the Sinovac-CoronaVac) were the first approved for mass vaccination. Although vaccines are widely available and vaccination rates are rising, people remain reluctant to get vaccinated immediately. Following a number of severe cases of blood clots after vaccination, some European countries have temporarily suspended AstraZeneca (AZD1222) vaccinations either fully or partially due to fear regarding thrombosis (2). Therefore, there is an urgent need to monitor and evaluate the safety of post-marketing vaccines as soon as possible.

It has been found that COVID-19 produces more severe symptoms and higher mortality among men than among women (3). Given the natural differences between two genders, women have stronger immune responses to foreign antigens and to self-antigens than men (4). Sex disparity should be taken into account in treatment and vaccine development. According to experience in monitoring adverse reactions to vaccines, many vaccines have shown sex differences (5, 6). Women tend to be more sensitive to vaccine reactions and are more prone to frequent and severe adverse effects. However, few studies to date have reported adverse events by sex, and very few analyses have evaluated sex differences in regard to the safety of SARS-CoV-2 vaccines.

Our previous real-world observational study indicated that the CoronaVac vaccine is safe because of the low proportion of self-reported adverse reactions (7). In this study we aimed to further explore sex-specific differences in adverse reactions to the vaccine, and to identify potential factors related to adverse reactions.

## Materials and Methods

### Study Design and Population

A cross-sectional survey was conducted among medical staff via the largest online survey platform (Wen-Juan-Xing) in China from 24 February to 7 March 2021. The inclusion criteria were all health professionals and administrative support staff aged 18–60 years who worked and were vaccinated with the Sinovac-CoronaVac COVID-19 vaccine at a tertiary hospital. Staff with an allergic constitution, neurological disorders including seizures and encephalopathy, severe chronic disease, or immunodeficiency disease as well as lactating/pregnant women were excluded. The details of the study design have previously been described (4). The interviewees received an invitation message or email to participate in the survey once or twice. Among the 3013 staff who completed their vaccination with two doses, a total of 1,397 interviewees responded to the questionnaire, including 1107 (79.2%) women and 290 (20.8%) men. The response rate was 46.4%. This study was exempted from informed consent and was approved by the Ethics Committee of Taizhou Hospital of Zhejiang Province (Approval number: K20210217) in China. All procedures were performed in accordance with the guidelines of our institutional ethics committee and adhered to the tenets of the Declaration of Helsinki. All participants' information was anonymous.

### Questionnaire

The structured questionnaire used in this study was shown in the Supplementary Material. Solicited and unsolicited local reactions and systemic adverse events during the period of one week following vaccination were collected. Local adverse reactions included adverse reactions at the injection site, such as pain, induration, redness, swelling, or itching. Solicited systemic adverse reactions included muscle pain, fatigue, headache and/or dizziness, fever, vomiting, diarrhea, appetite impairment, nausea, allergic reaction, urticaria, rash, severe fever, lymphadenopathy, cough, throat pain, stuffy, and runny nose. The unsolicited adverse reactions included other adverse reactions with an open-text response option in the questionnaire, such as menstruation, chest pain, and numbness of limbs, which were absent or seldom in the manual. Knowledge of the inactivated vaccine being used in the hospital was measured by the following question: “What platform of the COVID-19 vaccine do you think is being used in our hospital?” Attitudes towards the COVID-19 vaccine were tested by the questions “If conditions permit, will you take the COVID-19 vaccine for your family proactively?” and “Are you concerned about adverse effects of the COVID-19 vaccine?” History of adverse reactions to other vaccines, influenza vaccination, allergies, and underlying diseases was classified as yes or no. Health status and sleep quality before vaccination were categorized as good or bad. Positions were grouped into health professionals (doctors, nurses, medical technicians or pharmacists) and administrative support staff. Overweight and obesity were defined as a BMI greater than or equal to 24 kg/m^2^ according to Chinese criteria.

### Statistical Analysis

Categorical variables of adverse reactions and basic characteristics are displayed as counts and percentages in women and men; the chi-square test was used to initially assess possible influencing factors of adverse reactions post vaccination for each sex. Multinomial logistic regression is an extension of the (binary) logistic regression if the categorical dependent outcome has more than two levels. This model was then applied to identify the influencing factors of adverse effects, and the odds ratio (OR) and 95% confidence interval (CI) were calculated. All data were analyzed using IBM SPSS Statistics software (version 22.0; SPSS Inc., Chicago, IL, USA). A *p*-value of <0.05 was considered to represent a statistically significant difference among test populations.

## Results

### Basic Characteristics of the Study Population

Of the 3,013 vaccinated medical staff, 1,397 completed the questionnaire for a response rate of 46.4%. The respondents included 1,107 (79.2%) women and 290 (20.8%) men. The proportions of administrative support staff (31.0 vs. 20.4%, *P* < 0.001) and graduates (27.2 vs. 5.2%, *P* < 0.001) were higher in men than in women. There were also larger proportions of overweight (56.6 vs. 20.9%, *P* < 0.001), underlying diseases (19.7 vs. 7.9%, *P* < 0.001), and taking medication before vaccination (11.0 vs. 4.3%, *P* < 0.001) among men than among women. However, men were more likely to report good health status (96.2 vs. 91.1%, *P* = 0.004) and sleep quality before vaccination (81.0 vs. 75.2%, *P* = 0.039), and have positive attitudes towards vaccination for their family proactively (80.7 vs. 70.6%, *P* = 0.001) than women. Women were younger (mean age: 34.7 ± 8.6 years vs. 38.7 ± 9.9 years, *P* < 0.001) and more likely to worry about adverse reactions (57.4 vs. 41.0%, *P* < 0.001) than men.

### Sex Differences in Adverse Reactions Post Vaccination

As shown in Table 1, a total of 474 adverse reactions after the first dose were reported in 178 (16.1%) women, and 381 adverse reactions after the second dose were reported in 169 (15.3%) women. A total of 51 adverse reactions after the first dose were reported in 23 (7.9%) men, and 76 adverse reactions after the second dose were reported in 35 (12.1%) men. The most common adverse reaction was localized pain at the injection site, which accounted for 65.2% of the first adverse reactions and 73.4% of the second adverse reactions in women. The corresponding figures were 56.5 and 71.4%, respectively, in men. The most common systemic adverse reactions post-vaccination were muscle pain, fatigue, and headache and/or dizziness, with higher frequencies in women than in men. Sex differences were also observed for other solicited and non-solicited adverse reactions. All adverse reactions were mild and transient (Table 1).

**Table 1:** Distribution of multiple types of adverse reactions after vaccination in women and men.

**Adverse reactions**	**Women (** ***n*** **=** **1107)**						**Men (** ***n*** **=** **290)**					
	**After first dose**			**After second dose**			**After first dose**			**After second dose**		
	**No**.	**Frequency (%)**	**Proportion (%)**	**No**.	**Frequency (%)**	**Proportion (%)**	**No**.	**Frequency (%)**	**Proportion (%)**	**No**.	**Frequency (%)**	**Proportion (%)**
**Total adverse reactions**	178	16.1	100	169	15.3	100	23	7.9	100	35	12.1	100
**Solicited adverse reactions**												
**Injection site adverse reactions** (Pain,induration,redness,swelling, or itch)	116	10.5	65.2	124	11.2	73.4	13	4.5	56.5	25	8.6	71.4
**Systemic adverse reactions**												
Muscle pain	98	8.9	55.1	93	8.4	55.0	10	3.4	43.5	16	5.5	45.7
Fatigue	96	8.7	53.9	78	7.0	46.2	9	3.1	39.1	13	4.5	37.1
Headache, Dizziness	69	6.2	38.8	40	3.6	23.7	7	2.4	30.4	8	2.8	22.9
Fever	29	2.6	16.3	9	0.8	5.3	3	1.0	13.0	5	1.7	14.3
Vomiting, Diarrhea	16	1.4	9.0	9	0.8	5.3	3	1.0	13.0	4	1.4	11.4
Appetite impaired, Nausea	15	1.4	8.4	11	1.0	6.5	0	0	0	2	0.7	5.7
Allergic reaction, urticaria, rash	10	0.9	5.6	0	0	0	2	0.7	8.7	0	0	0
Stuffy, runny nose	9	0.8	5.1	5	0.5	3.0	1	0.3	4.3	0	0	0
Cough, Throat pain	8	0.7	4.5	6	0.5	3.6	2	0.7	8.7	1	0.3	2.9
Lymphadenopathy	2	0.2	1.1	3	0.3	1.8	1	0.3	4.3	1	0.3	2.9
**Non-solicited adverse reactions**												
(Menstruation, chest pain, numbness of limbs)	6	0.5	3.4	3	0.3	1.8	0	0	0	1	0.3	2.9

### Factors Associated With Adverse Reactions in Women and Men

Table 2 indicates the sex-specific frequencies of adverse effects after one or two vaccinations using univariate analysis. In women, a history of adverse reactions to other vaccines, concerns about adverse reactions, knowledge of the inactivated vaccine being used in the hospital, taking vaccines for family proactively, receiving an influenza vaccination, a history of allergic reactions, health status, and sleep quality before vaccination were significant factors affecting adverse reactions after one or two inoculations. In contrast, overweight, underlying disease, a history of adverse reactions to other vaccines, and concerns regarding adverse reactions were associated with the risk of adverse reactions to vaccination in men.

**Table 2:** Univariate analysis of associated factors for adverse reactions in women and men.

**Variables**	***n***	**Adverse reactions in women (** ***n*** **=** **1107)**					***n***	**Adverse reactions in men (** ***n*** **=** **290)**				
		**After one vaccination**		**After both vaccination**				**After one vaccination**		**After both vaccination**		
		**No**.	**Frequency (%)**	**No**.	**Frequency (%)**	***P***		**No**.	**Frequency (%)**	**No**.	**Frequency (%)**	***P***
**Age (years)**						0.237						0.475
18-29	346	48	13.9	29	8.4		71	8	11.3	4	5.6	
30-39	482	81	16.8	42	8.7		77	8	10.4	7	9.1	
40-49	231	25	10.8	17	7.4		97	9	9.3	2	2.1	
50-60	48	11	22.9	3	6.3		45	5	11.1	1	2.2	
**Total service time (years)**						0.863						0.924
<5	373	58	15.5	29	7.8		106	10	9.4	5	4.7	
≥5	734	107	14.6	62	8.4		184	20	10.9	9	4.9	
**Education level**						0.186						0.194
Junior College and below	359	50	13.9	21	5.8		87	6	6.9	1	1.1	
Undergraduate	690	104	15.1	63	9.1		124	15	12.1	7	5.6	
Graduate	58	11	19.0	7	12.1		79	9	11.4	6	7.6	
**Position**						0.158						0.605
Health professionals	881	136	15.4	78	8.9		200	22	11.0	11	5.5	
Administrative support staff	226	29	12.8	13	5.8		90	8	8.9	3	3.3	
**Professional titles**						0.813						0.406
Internship or primary	466	73	15.7	37	7.9		109	8	7.3	6	6.0	
Medium or higher	641	92	14.4	54	8.4		181	22	12.2	8	4.4	
**Body mass index (kg/m** ^**2**^ **)**						0.148						**0.031**
<24	876	139	15.9	68	7.8		126	10	7.9	2	1.6	
≥24	231	26	11.3	23	10.0		164	20	12.2	12	7.3	
**Underlying disease**						0.186						**0.032**
No	1020	148	14.5	81	7.9		233	20	8.6	9	3.9	
Yes	87	17	19.5	10	11.5		57	10	17.5	5	8.8	
**Take medication before vaccination**						0.052						0.682
No	1059	153	14.4	85	8.0		258	28	10.9	12	4.7	
Yes	48	12	25.0	6	12.5		32	2	6.3	2	6.3	
**Adverse reactions to other vaccines**						**<0.001**						**<0.001**
No	1046	140	13.4	81	7.7		277	28	10.1	8	2.9	
Yes	61	25	41.0	10	16.4		13	2	15.4	6	46.2	
**Worry about adverse reactions**						**<0.001**						**<0.001**
No	472	41	8.7	24	5.1		171	6	3.5	7	4.1	
Yes	635	124	19.5	67	10.6		119	24	20.2	7	5.9	
**Knowledge of inactivated vaccine being used in the hospital**						**0.015**						0.266
Inactivated vaccine	874	138	15.8	80	9.2		232	26	11.2	13	5.6	
Others	233	27	11.6	11	4.7		58	4	6.9	1	1.7	
**Take vaccine for the family proactively**						**<0.001**						0.274
Yes	782	98	12.5	52	6.6		234	24	10.3	9	3.8	
No/Not sure	325	67	20.6	39	12.0		56	6	10.7	5	8.9	
**Get influenza vaccination**						**0.021**						0.913
No	802	114	14.2	56	7		217	23	10.6	11	5.1	
Yes	305	51	16.7	35	11.5		73	7	9.6	3	4.1	
**Allergic reaction**						**0.008**						0.252
No	1038	146	14.1	85	8.2		275	28	10.2	12	4.4	
Yes	69	19	27.5	6	8.7		15	2	13.3	2	13.3	
**Health status before vaccination**						**<0.001**						0.795
Good	1009	134	13.3	78	7.7		279	29	10.4	13	4.7	
General /Worse	98	31	31.6	13	13.3		11	1	9.1	1	9.1	
**Sleep quality before vaccination**						**<0.001**						0.248
Good	833	98	11.8	58	7.0		235	21	8.9	11	4.7	
General /Worse	274	67	24.5	33	12.0		55	9	16.4	3	5.5	

*Statistically significant values at p < 0.05 are shown in bold*.

The effect of independent associated risk factors on each type of adverse reaction was examined using a multinomial logistic regression model. As depicted in Table 3, after adjustment for confounding factors, adverse reactions to other vaccines (yes vs. no, OR = 4.42, 95%CI: 2.39–8.18), worry about adverse reactions (yes vs. no, OR = 2.14, 95%CI: 1.41–3.23), knowledge of the inactivated vaccine being used in the hospital (yes vs. no, OR = 0.58, 95%CI: 0.36–0.93), allergic history (yes vs. no, OR = 2.29, 95%CI: 1.25–4.19), health status before vaccination (general/worse vs. good, OR = 1.90, 95%CI: 1.11–3.24), and sleep quality before vaccination (general/worse vs. good, OR = 1.81, 95%CI: 1.23–2.67) were significantly related to adverse reactions from vaccination; adverse reactions to other vaccines, worry about adverse reactions, knowledge of the inactivated vaccine being used in the hospital, taking the vaccine for one's family proactively, and getting an influenza vaccination were significantly related to adverse reactions to both injections in women. In contrast, concerns regarding adverse reactions independently increased the risk of adverse reactions following vaccination (OR = 6.79, 95% CI: 2.66–17.37), and a history of adverse reactions to other vaccines increased the risk of adverse reactions to both injections in men (OR = 31.30, 95% CI: 7.35–133.35). In addition, there was a borderline association between overweight and adverse reactions to both injections in men (OR = 4.09, 95% CI: 0.80–20.98, *P* = 0.091).

**Table 3:** Multinominal logistic regression of associated factors for adverse reactions in women and men.

**Variables**	**Adverse reaction in any vaccination**			**Adverse reaction in both vaccination**		
	**vs**.			**vs**.		
	**No adverse reaction**			**No adverse reaction**		
	***OR***	**95% *CI***	***P***	***OR***	**95% *CI***	***P***
**Women (** ***n*** **=** **1107)**						
Adverse reactions to other vaccines (yes vs. no)	4.42	2.39–8.18	**<0.001**	3.16	1.42–7.02	**0.005**
Worry about adverse reactions (yes vs. no)	2.14	1.41–3.23	**<0.001**	2.01	1.19–3.38	**0.009**
Knowledge of inactivated vaccine being used in the hospital (yes vs. no)	0.58	0.36–0.93	**0.024**	0.42	0.22–0.81	**0.010**
Take vaccine for family proactively (yes vs. No or not sure)	0.70	0.48–1.03	0.073	0.61	0.37–0.98	**0.043**
Get influenza vaccination (yes vs. no)	1.19	0.80–1.75	0.391	1.73	1.09–2.75	**0.020**
Allergic history (yes vs. no)	2.29	1.25–4.19	**0.007**	1.22	0.49–3.01	0.671
Health status before vaccination (General/Worse vs. Good)	1.90	1.11–3.24	**0.019**	1.59	0.78–3.24	0.200
Sleep quality before vaccination (General/Worse vs. Good)	1.81	1.23–2.67	**0.003**	1.56	0.95–2.57	0.081
**Men (** ***n*** **=** **290)**						
Adverse reactions to other vaccines (yes vs. no)	2.55	0.43–14.96	0.299	31.30	7.35–133.35	**<0.001**
Worry about adverse reactions (yes vs. no)	6.79	2.66–17.37	**<0.001**	1.19	0.34–4.16	0.781
Body mass index (overweight vs. non-overweight)	1.34	0.57–3.14	0.507	4.09	0.80–20.98	0.091
Underlying disease (yes vs. no)	2.11	0.87–5.13	0.100	1.99	0.55–7.24	0.294

*Statistically significant values at p < 0.05 are shown in bold*.

## Discussion

### Clinical Implications

To the best of our knowledge, although previous studies have indicated the disparity of sex in adverse reactions to a number of vaccines (8, 9), few studies have assessed sex-specific differences in adverse reactions to an inactivated SARS-CoV-2 vaccine in active surveillance. This study was conducted with 1,107 women and 290 men who worked at a hospital in China during the period of emergency use of the vaccine. Our findings showed that the frequency of overall adverse reactions was higher in women than in men, regardless of whether it was the first or second injection. The most common adverse reaction was localized pain at the injection site, followed by muscle pain, fatigue, and headache and/or dizziness in both women and men. Almost all types of adverse reactions were more common in women than in men. These findings related to sex differences were similar to those observed regarding other vaccines against influenza, hepatitis B, and yellow fever (10). A systematic review published in 2019 also showed a higher frequency of adverse events in women after influenza vaccination (5), particularly local reactions. A real-world study based on the national post-marketing surveillance data for the Pfizer-BioNTech and Moderna COVID-19 vaccines in the United States found that more females reported adverse events following COVID-19 vaccination, compared to males, but males were more likely to experience serious adverse events, death, and hospitalization than females (11). More women developed anaphylaxis reactions to the Pfizer-BioNTech vaccine in U.S. (12, 13), UK (14) and Japan (15). The sex disparity of adverse reactions was also observed following AstraZeneca (AZD1222) Vaccine or BNT162b2 COVID-19 Vaccine in South Korea (16, 17). A retrospective descriptive study using spontaneous reports showed that thrombotic adverse reactions were associated with the COVID-19 AstraZeneca vaccine, in which approximately double the number of occurrences of potential thrombotic events reported in women (*n* = 19) than men (*n* = 9) (18). In contrast, a cross-sectional study in Saudi Arabia showed that men were more likely to report fever, skin rash, and pain at the injection site following the first dose of the AstraZeneca (AZD1222) COVID-19 vaccine (19). Therefore, sex disparity in adverse reactions to vaccines may be related to the type of vaccine and the severity of the adverse reaction.

### Mechanism of Sex Differences in Vaccine Response

The pathophysiology of sex differences in adverse reactions following immunization is multifactorial. Clear biological differences between the sexes can usually be attributed to immunological, hormonal, or genetic factors or a combination of the three. Adult females tend to have stronger inflammatory responses to vaccines than males, and these differences may result in both the female-biased efficacy of vaccines and female-biased adverse events following vaccination. Sex hormones modulate the function of immune cells, including β cells, which results in differential immune responses between the sexes. Oestrogens have immune-suppressive effects at higher levels and immune-stimulant activity at lower levels (20, 21), while testosterone suppresses innate immune responses at all times. Females have two X chromosomes which carry many genes related to immune mechanisms, while males just have one. Angiotensin converting enzyme 2 (ACE2), a functional receptor for SARS-CoV-2, is encoded by its homologous gene (ACE2), which maps on chromosome X (Xp2.22) (22). Recent research has demonstrated that the angiotensin-converting enzyme-2 (ACE2) receptor is an essential port of cell entry for the spike (S) protein of SARS-CoV-2 (23). It has been reported that estrogen (17β-estradiol) inhibits ACE2 activity, but androgen upregulates the activity of ACE2 (24, 25).

Beyond the biological differences, a growing body of evidence supports gender-based social/behavioral differences in vaccine response, such as different comorbidity rates (e.g. obesity, hypertension, and cardiovascular diseases), smoking and drinking habits, educational levels, and societal roles (10). A recent study demonstrated that the expression of ACE2 increases under cigarette smoke exposure and inflammatory stimulation (26). The smoking rate is typically higher among men than among women (50.5 vs. 2.1%) according to the 2018 Global Adult Tobacco Survey (27). The prevalence of obesity among men was 2.7 times higher than that among women in this study. Women constitute the majority of nurses and caregivers both in hospitals and within families, with 70% of the world's healthcare staff is composed of women. Women were more likely to perceive and/or report adverse reactions compared to men, leading to a higher response rate (28). In addition, social culture contexts, such as Confucian morals and socialistic norms, will be also related to disparities in health-seeking behavior and reporting adverse reactions between men and women (29, 30). Despite this recognition, there is little evidence demonstrating these effects. Further research is needed to clarify whether the observed sex differences are primarily due to a disproportionate share of unhealthy behavior between genders or are related to different immune responses or other factors.

### Methodological Considerations

The main strengths of our study include the real-world design to better reflect real life, data collection using an active surveillance method, and very limited missing data. Sample representativeness was the main limitation of our study. The sample was recruited from only one hospital, and the participants were likely to be younger and healthier than the general population. The majority of respondents were female. The selected bias may have resulted in an overestimation of the proportion of adverse reactions. Potential report and recall bias should also be noted, although all participants had been vaccinated within 7 weeks prior to completing the survey. Data were collected using self-administered questionnaires. No assessment was made as to whether the reported adverse reactions were related to vaccination. Finally, our measurements were performed at only a single point in time. We were unable to validate the anonymous survey. Moreover, this study does not reflect long-term exposure to factors that may be important for adverse reactions to the inactivated SARS-CoV-2 vaccine.

## Conclusions

In conclusion, our study implies that the frequency of reported adverse reactions to the inactivated SARS-CoV-2 vaccine was higher in women than in men, with more potential factors related to vaccine responses in women in a sample of medical staff. Further studies are needed to determine the underlying factors and mechanisms of sex differences in regard to adverse reactions. The female-biased adverse reactions may introduce worry about adverse reactions, leading to more vaccine hesitancy in women. More attention should be paid to the vaccine acceptance in the female population during mass vaccination.

## Data Availability Statement

The raw data supporting the conclusions of this article will be made available by the authors, without undue reservation.

## Ethics Statement

The studies involving human participants were reviewed and approved by the Ethics Committee of Taizhou Hospital of Zhejiang Province in China (Reference No. K20210217, approved on 26 February 2021). Written informed consent for participation was not required for this study in accordance with the national legislation and the institutional requirements. Written informed consent was not obtained from the individual(s) for the publication of any potentially identifiable images or data included in this article.

## Author Contributions

J-SZ and T-HT: conceptualization. M-XZ and T-HT: methodology. M-XZ, W-YY, and G-FS: investigation. M-XZ and G-FS: data curation. M-XZ: formal analysis and writing—original draft preparation. SQ and T-HT: validation. T-HT: writing—review, editing, visualization, and project administration. C-WC and H-XC: supervision. All authors have read and agreed to the published version of the manuscript.

## Conflict of Interest

The authors declare that the research was conducted in the absence of any commercial or financial relationships that could be construed as a potential conflict of interest.

## Publisher's Note

All claims expressed in this article are solely those of the authors and do not necessarily represent those of their affiliated organizations, or those of the publisher, the editors and the reviewers. Any product that may be evaluated in this article, or claim that may be made by its manufacturer, is not guaranteed or endorsed by the publisher.
