# Supplementary material for: Sex Differences in Adverse Reactions to an Inactivated SARS-CoV-2 Vaccine Among Medical Staff in China
Source: Front Med (Lausanne). 2021 Sep 8;8:731593. doi: 10.3389/fmed.2021.731593 (PMC8455952; doi:10.3389/fmed.2021.731593)
Supplement: Supplementary file 1 [file Data_Sheet_1.pdf]

Questionnaire on the Safety of the COVID-19 Vaccine among Medical Staff

This is a questionnaire regarding adverse reactions to the COVID-19 vaccine and the possible influencing factors. The data collected by the questionnaire were only used for statistical analysis. This study was approved by the ethics committee. You have volunteered to participate in this survey.

- **Basic Information**

1. **Gender:** □1=male □2=female
2. **Date of Birth:** □□□□year□□month□□day
3. **Education Level:**

   □1=Junior College and below □2=Undergraduate □3=Graduate
4. **Position:** □1=Health professionals □2= Administrative support staff
5. **Professional titles:** □1= Internship or primary □2= Medium or higher
6. **Total service time (years):** □□
7. **Height:** □□□cm
8. **Weight:** □□□kg
9. **Are there any underlying diseases prior to vaccination against COVID-19?**

   □0 = None

   □1 = Hypertension

   □2 = Diabetes

   □3 = Dyslipidaemia

   □4 = Gout

   □5 = Heart disease

   □6 = Cerebrovascular disease

   □7 = Cancer /malignant tumours

   □8 = Other diseases

10. Did you take medicine before receiving the COVID-19 vaccine?

□1=Yes □2=No

11. Have you had any adverse reactions to other vaccines?

□1=Yes □2=No

12. Are you concerned about adverse effects of the COVID-19 vaccine?

□1=Yes □2=No

13. What platform of the COVID-19 vaccine is being used in our hospital?

□1=RNA vaccine □2=Inactivated vaccine □3=Viral vector vaccine

□4= Protein/virus-like particle vaccine □5=Other

14. If conditions permit, will you take the COVID-19 vaccine for your family proactively?

□1=Yes □2=No □3=Not sure

15. Have you ever been vaccinated against influenza?

□1=Yes □2=No

16. Have you ever had a severe allergic reaction to drugs, food, pollen, etc. ?

□1=Yes □2=No

17. Health status before vaccination with the COVID-19 vaccine:

□1=Good □2=Moderate □3=Bad

18. Sleep quality before vaccination with the COVID-19 vaccine:

□1=Good □2=Moderate □3=Bad

- **Adverse Reaction to the COVID-19 Vaccine**

19. How many doses of the COVID-19 vaccine have you been vaccinated with?

□0=Unvaccinated □1=Vaccinated 1 dose □2= Vaccinated 2 doses

20. Did you experience the following adverse reactions after receiving the first and second doses of the COVID-19 vaccine? □1=Yes □2=No

**Adverse reactions**	**After first dose**	**After second dose**
1) Fever	☐	☐
2) Fever over 38.5℃, repeated fever, or fever lasting more than 48 hours	☐	☐
3) Pain, induration, redness, swelling, or itchiness at the injection site	☐	☐
4) Allergic reaction, urticaria, rash	☐	☐
5) Fatigue	☐	☐
6) Muscle pain	☐	☐
7) Headache, dizziness	☐	☐
8) Appetite impaired, nausea	☐	☐
9) Vomiting, diarrhoea	☐	☐
10) Stuffy, runny nose	☐	☐
11) Cough, throat pain	☐	☐
12) Lymphadenopathy	☐	☐
13) Other adverse reactions	☐	☐
	____________	____________
	____________	____________
	____________	____________

--The End--
